# Interface Joint Strength between SS316L Wrought Substrate and Powder Bed Fusion Built Parts

**DOI:** 10.3390/ma14113041

**Published:** 2021-06-03

**Authors:** Jason M. Weaver, John R. Linn, Michael P. Miles

**Affiliations:** Department of Manufacturing Engineering, Brigham Young University, Provo, UT 84602, USA; johnrosslinn@gmail.com (J.R.L.); mmiles@byu.edu (M.P.M.)

**Keywords:** powder bed fusion, additive manufacturing, ss316l, interface strength

## Abstract

Metal powder bed fusion (PBF) additive manufacturing (AM) builds metal parts layer by layer upon a substrate material. The strength of this interface between the substrate and the printed material is important to characterize, especially in applications where the substrate is retained and included in the finished part. Ensuring that this interface between the original and the printed material has adequate material properties enables the use of this PBF AM process to repair existing structures and create new parts using both AM and conventional manufacturing. This paper studies the tensile and torsional shear strengths of wrought and PBF-built SS316L specimens and compares them to specimens that are composed of half wrought material and half PBF material. These specimens were created by building new material via PBF onto existing wrought SS316L blocks, then cutting the specimens to include both materials. The specimens are also examined using optical microscopy and electron backscatter diffraction (EBSD). The PBF specimens consistently exhibited higher strength and lower ductility than the wrought specimens. The hybrid PBF/wrought specimens performed similarly to the wrought material. In none of the specimens did any failure appear to occur at or near the interface between the wrought substrate and the PBF material. In addition, most of the deformation in the PBF/wrought specimens appeared to be limited to the wrought portion of the specimens. These results are consistent with optical microscopy and EBSD showing smaller grain size in the PBF material, which correlates to increased strength in SS316L due to the Hall–Petch relationship. With the strength at the interface meeting or exceeding the strength of the original wrought material, this process shows great promise as a method for adding additional features or repairing existing structures using metal PBF AM.

## 1. Introduction

The additive manufacturing (AM) of metals can currently be accomplished through several different methods. Two common technologies in industrial settings are powder bed fusion (PBF) and directed energy deposition (DED). In PBF processes, metal powder is spread evenly over a flat surface and selectively welded or sintered together using a laser, electron beam, or other energy source [1]. In DED processes, metal particles are fed into a melt pool on a work surface and simultaneously welded together, again using a laser, electron beam, or other energy source [2]. In both PBF and DED, the fused metal particles build upon an existing substrate material. The desired part is usually distinct from the substrate and is cut off during post-processing. However in certain applications, the substrate remains an integral part of the finished product [3]. This paper examines the feasibility of using PBF in these situations.

There are several scenarios where the ability to print onto existing structures is beneficial. One application of interest is the capability to build new features onto a part that has been manufactured through other means [4,5]. Many products (such as aircraft engine turbines [6], and oil and gas machinery [7,8]) consist of features with complex geometries attached to a central body with a simple geometry (such as a cylindrical shaft). Using AM to print the complex features can lead to substantial savings in machining costs and material waste, while also allowing greater design flexibility. However, it would be inefficient and expensive to also print the simple shaft or hub using AM. Printing the AM features separately and welding or fastening them to the core structure can be undesirable due to the reduced strength and the added assembly time and complexity. If the complex features can be printed directly onto a conventionally manufactured shaft or hub, a superior product and overall manufacturing process is possible [9]. Similarly, dies with integrated conformal cooling channels can be created by utilizing conventional methods for the main bodies of the dies, then printing the cooling channel structure onto the main bodies [10].

Another scenario of interest is the remanufacturing or repair of high-wear parts that would be expensive to replace [4,11,12]. For example, if a single turbine blade in an aircraft engine becomes damaged and cannot be repaired, either the blade must be removed and replaced, or even worse, the entire turbine must be replaced. If instead, the damage can be repaired by filling the damaged area using metal AM processes and then machining the new material to the required tolerances, the original part can still be used [13,14,15].

Both PBF and DED are viable options for printing onto an existing part as the substrate [16,17]. DED is often the AM process of choice for applications where the substrate is an existing part, as it has much greater freedom in building from non-horizontal or complex surfaces [18]. PBF processes are currently more generally available in the industry and often have a better accuracy and surface finish [19], but any substrate surface being printed on must be planar and secured horizontally within the powder bed [20]. Due to of this limitation, the use of PBF to print onto existing structures that are retained in the final part is not common in the industry, though recent studies have begun considering this as an option [10,14,21].

For PBF to be more widely used in these applications, the material characteristics at the interface between the original substrate material and the new AM material must be better understood [10,16,22]. Ideally, the characteristics at the interface should be identical to or better than the characteristics of the original material and the printed material. If the interface does exhibit suboptimal characteristics, these should be compared to those of the original material and printed material to determine if the proposed processes are acceptable solutions for building hybrid AM/conventional parts.

Recent studies have examined various material properties near the substrate interface of PBF materials, specifically maraging steels and titanium alloys. Azizi et al. [10] examined maraging steel powder printed using PBF onto both C300 maraging steel and H13 tool steel substrates. They found that both hybrid combinations yielded tensile properties similar to the wrought materials, especially following heat treatment. They also found that while the maraging/maraging specimens tended to fail in the AM printed material (they hypothesized that this was due to porosity between the layers), the maraging/tool steel specimens tended to fail at the interface (due to both porosity and chemical/microstructural inhomogeneity). Shakerin et al. [23] also studied the combination of maraging steel printed onto a H13 tool steel substrate. They also found an abrupt change in the microstructure at the interface, without much of an alteration in the grain structure of the substrate, but they did not find significant porosity. They determined that the printed material was harder than the wrought tool steel, and that the hardness increased around the interface on the AM printed side. Tensile failures in their specimens tended to occur in the tool steel substrate, far away from the interface. Dolev et al. [22] studied Ti-6Al4-V printed onto a substrate of the same material. They found that their specimens also tended to fracture in the wrought substrate material, and that most of the deformation was concentrated there as well. They found that the AM printed material showed a similar strength to the wrought material but that it was initially much less ductile. However, heat treatment increased the ductility of the AM material to near that of the wrought material, without substantially reducing its tensile strength. All three papers concluded that the use of these materials was viable for the intended hybrid AM/wrought applications.

This paper characterizes the tensile strength, torsional shear strength, and grain size at or near the interface of test specimens built from SS316L, using a PBF AM process. SS316L is a common stainless steel that is frequently used in both conventional fabrication and in PBF AM applications. A better understanding of the interface between the substrate and the printed material will allow for the wider use of this steel in AM repair and hybrid manufacturing. Similar to the studies described above, this study is intended to determine whether hybrid AM/wrought parts of SS316L can perform with similar properties to the parts made wholly from wrought material.

## 2. Materials and Methods

A summary of the number and types of prepared specimens and completed tests is shown in Table 1. Specimens of wrought SS316L and AM-printed SS316L were compared to hybrid specimens of half-wrought, half-AM SS316L to evaluate the interface bond quality of the AM/wrought specimens. A 400W EOS M280 PBF-laser system (EOS GmbH, Krailling, Germany) was used to manufacture the AM and hybrid AM/wrought specimens from SS316L. Stock settings for the materials were used on the machine. No additional heat treatments took place following the fabrication.

**Figure 1:**
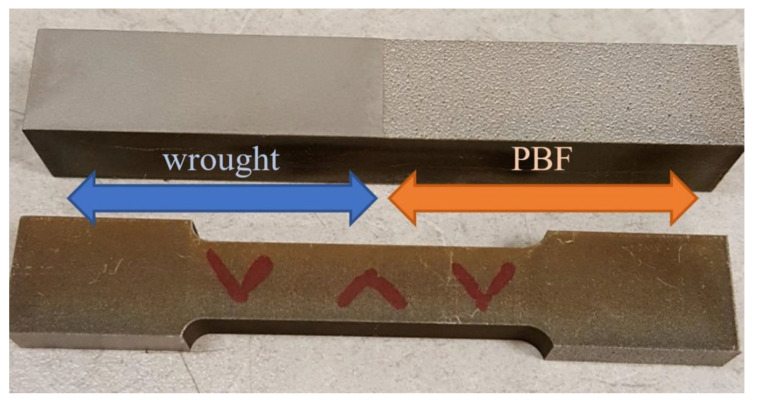
A 3-inch tensile specimen design (ASTM E8).

**Figure 2:**
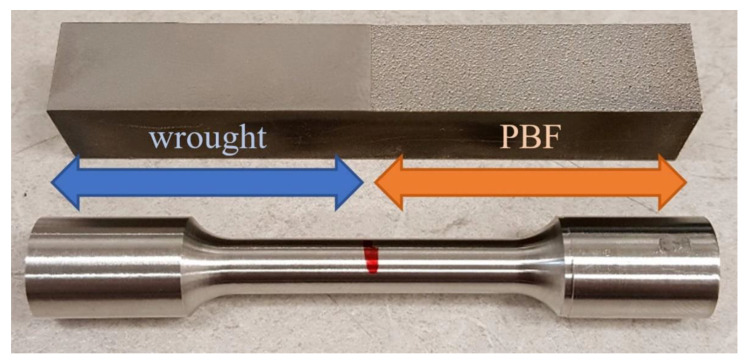
A 3-inch torsion specimen design (ASTM E8).

The blocks of wrought material and blocks produced using PBF were machined into the test specimens to evaluate the tensile and torsional responses, following ASTM E8 specifications [24]. Photographs of the sample PBF/wrought specimens are shown in Figure 1 and Figure 2.

The uniaxial tensile testing was performed on an Instron Model 1381 frame (Instron, Norwood, MA, USA), with an 8800 controller. The testing was conducted using the following parameters:

- Pull rate of 2 in/min
- Data acquisition at 10 Hz
- Measured: elongation (in) vs tensile load (lb)

The measured elongations and tensile loads were converted to strain and tensile stress in SI units using the standard formulas.

The torsion testing was performed on the same Instron machine, using the following parameters:

- Rotation rate of 6°/min
- Rotation range of 90°
- Data acquisition at 10 Hz
- Measured: rotation (deg) vs. torque load (lb-in)

The measured torque load and rotation were converted to maximum shear stress in SI units (using the formula *τ* = *T r/J*, where ***τ*** is the maximum shear stress, *T* is the measured torque load, *r* is the radius at the location of interest, and *J* is the polar moment of inertia for a cylinder, *π r^4^*/2) and rotation per unit length of the narrow section (in rad/m).

**Figure 3:**
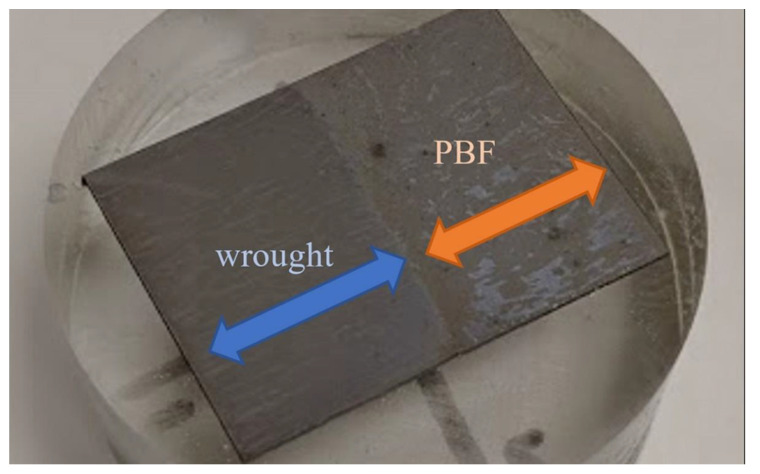
Etched PBF/wrought SS316L microscopy specimen.

A specimen was also prepared for visual analysis via microscopy. The microscopy specimen was cut on a wire EDM (electrical discharge machining) with the boundary between the PBF and wrought material centered on the face. The specimen was placed in an epoxy puck, ground progressively to 1200 grit sandpaper, polished with diamond paste and alumina, and then etched using a Carpenter 300 Series stainless steel etchant. The specimen was also prepared for electron backscatter diffraction (EBSD) by the polishing routine described above, followed by an electropolish in a perchloric acid–methanol solution and a polish with colloidal silica. The etched microscopy specimen is shown in Figure 3. The finished microscopy specimen was viewed under a microscope at 32× and 63× magnification. EBSD scans were performed using an S-FEG XL30 FEI microscope (ThermoFisher Scientific, Hillsboro, OR, USA). EBSD patterns were collected and processed using EDAX’s OIM Data Collection v. 7 (Ametek, Mahwah, NJ, USA).

## 3. Results

Below are the test results for tensile testing, torsional testing, and microscopy.

### 3.1. Tensile Testing

**Figure 4:**
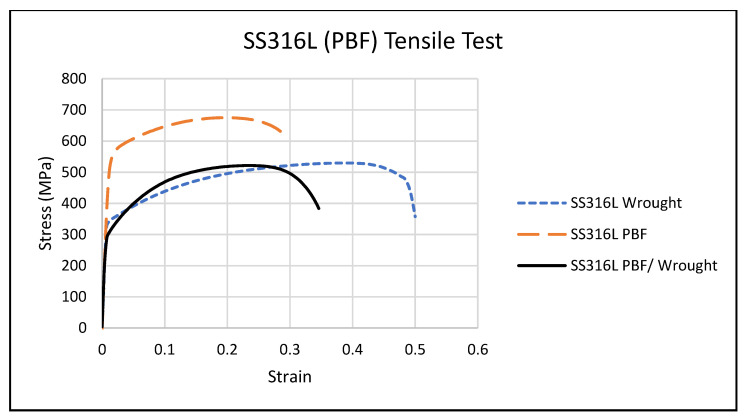
Average tensile test stress/strain.

The tensile testing results are shown in Figure 4, comparing the wrought and PBF/wrought specimens, where each curve represents an average of three specimens. Note that the stress and strain measured are the engineering (nominal) stress and strain. The wrought and PBF/wrought specimens performed similarly under elastic deformation, while the PBF specimens exhibited a much higher yield strength and ultimate tensile strength. Both the monolithic PBF and the PBF/wrought specimens failed at a lower strain than the wrought SS316L. After initial yielding, the PBF specimens failed at the lowest strain, followed by the PBF/wrought specimens and then the wrought specimens. The necking and failure in the PBF/wrought specimens occurred within the wrought material in each case.

Table 2 shows the average yield strength ($\sigma_{y}$) and ultimate tensile strength ($\sigma_{u}$) for the wrought, PBF, and PBF/wrought specimens. Table 3 shows the statistical analysis (α = 0.05) that the differences in $\sigma_{y}$ and $\sigma_{u}$ between the PBF specimens and the two other groups to be significant.

### 3.2. Torsional Testing

**Figure 5:**
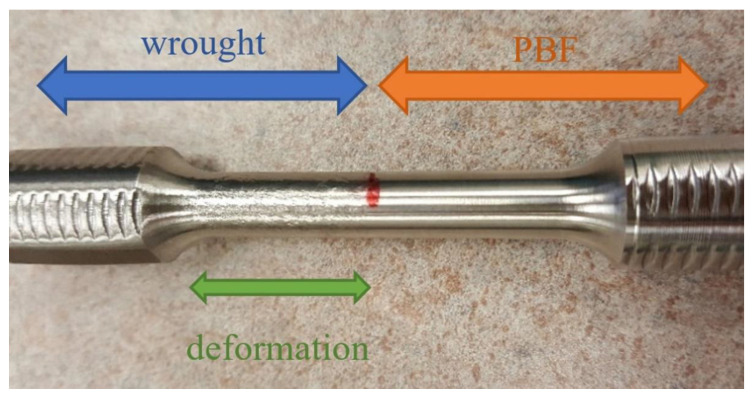
SS316L (PBF/wrought) torsion specimen post-failure.

The results of a torsion test are shown below in Figure 5, where the deformation is concentrated in the wrought portion of the specimen. This was the same tendency seen in the tensile specimens.

**Figure 6:**
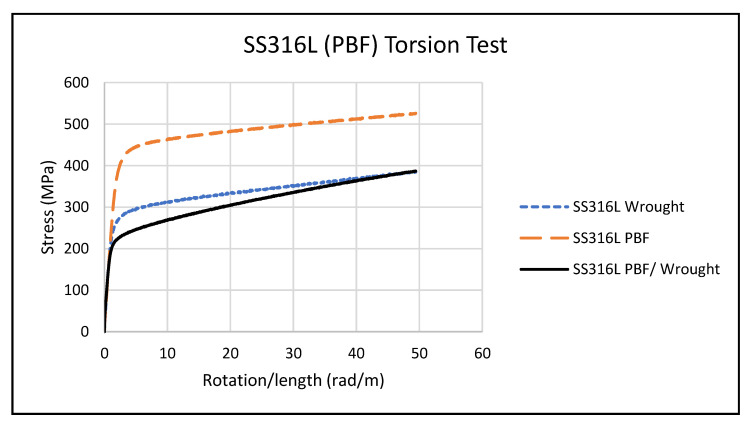
Average torsion test stress/rotation.

Figure 6 shows the average stress profiles of SS316L in torsion for the two wrought, three PBF, and three PBF/wrought specimens. The wrought and PBF/wrought specimens performed similarly, while the PBF specimens exhibited a much higher $\tau_{y}$. Table 4 shows the average torsional shear strength ($\tau_{y}$) and shear modulus (G) for the wrought, PBF, and PBF/wrought specimens.

Table 5 shows the statistical analysis (α = 0.05) that found the difference in $\tau_{y}$ between the PBF specimens and the other two groups to be significant. The differences in G between the groups was found to be barely significant using ANOVA but was not substantial enough to discern a significance between any two of the groups.

### 3.3. Microscopy

**Figure 7:**
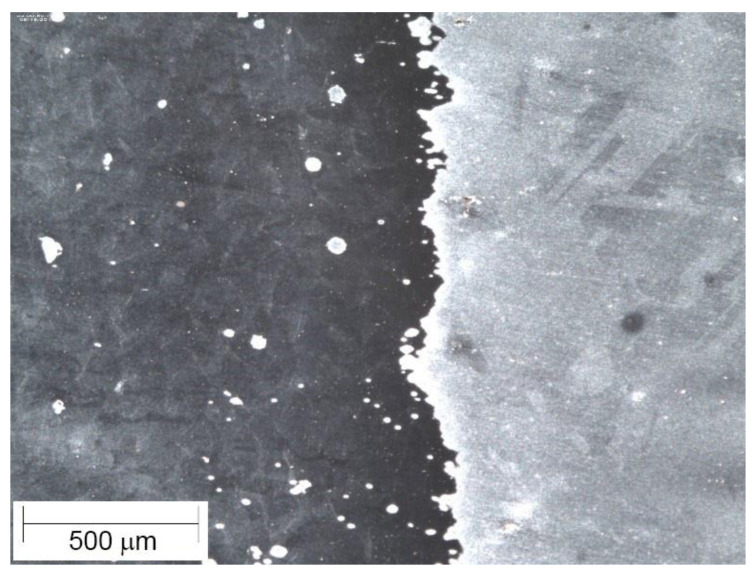
SS316L PBF/wrought interface (63× magnification).

Optical microscopy was used to examine the interface between the PBF and the wrought 316L, while also allowing for the characterization of grain size. Figure 7 shows a representative image of the interface in the PBF/wrought 316L specimen. Formal analysis of void content was not carried out, but the micrograph in Figure 7 (at 63× magnification) shows no evidence of voids at the interface or in the PBF material.

**Figure 8:**
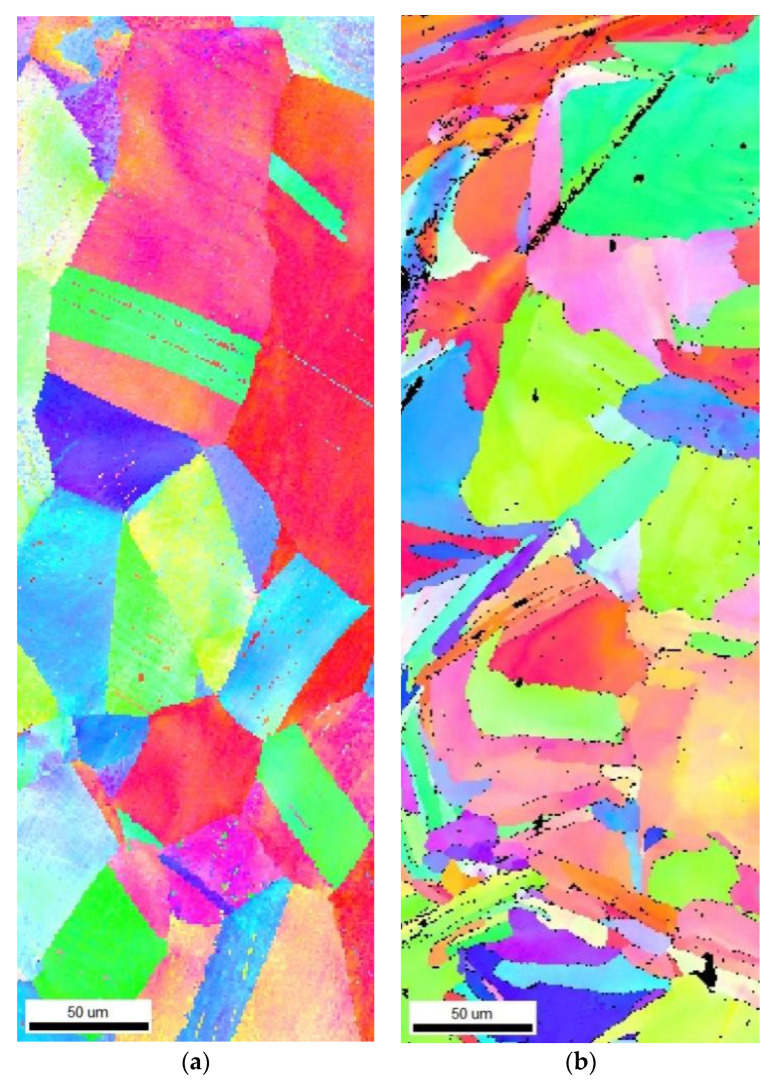
EBSD grain maps of (**a**) wrought SS316L and (**b**) SSPBF 316L. Scans were taken about 10 mm away from the PBF/wrought interface.

EBSD scans provided images of the grain structure and the average grain size calculations. Calculated grain sizes for the wrought and PBF materials are shown in Table 6, with grain maps for each material shown in Figure 8.

## 4. Discussion

From the results described above, we can determine several consistent characteristics among the wrought, PBF, and hybrid wrought/PBF specimens.

### 4.1. Analysis of Wrought and PBF AM Specimens

The tensile and torsion stress curves of the monolithic PBF AM material were higher than those of the wrought material. The measured tensile yield strength, ultimate tensile strength, and torsional shear strength of the PBF specimens were consistently greater than those of the wrought specimens, and plastic deformation occurred at higher stresses for given displacement levels. The PBF specimens also failed under tension at a lower strain, showing less ductility than the wrought material. EBSD analysis of the wrought and PBF materials supports the test results. The SS316L wrought material had an average grain size of 60 µm, while the PBF material had an average grain size of 48 µm. Since SS316L exhibits a very strong Hall–Petch relationship, where the yield stress increases with a smaller grain size [27], the smaller grains in the PBF material increase their strength over the wrought material. The smaller grain size in the PBF material also contributes to the reduced ductility, as the slip of the grains is more restricted.

### 4.2. Analysis of Hybrid PBF/Wrought Specimens

In all the tensile tests, the PBF/wrought specimens consistently experienced failure well away from the interface between the two materials. This demonstrates a good bond quality at the interface and strengthening of the weaker material by the stronger material near the interface. As no failures occurred at the interface, the actual interfacial strength could not be determined, other than the conclusion that it would be higher than the observed stresses at failure. As mentioned above, the PBF material exhibited a significantly higher strength than the wrought material, most likely due to its reduced grain size. In the hybrid PBF/wrought specimens, plastic deformation, and final tensile failure consistently occurred almost exclusively within the lower-strength wrought portions of the specimens. The measured tensile yield strength, ultimate tensile strength, and torsional yield strength of the PBF/wrought specimens were not significantly different from the baseline wrought specimens, supporting this observation.

## 5. Conclusions

Powder bed fusion (PBF) AM 316L stainless steel material was built up onto existing wrought 316L blocks, allowing for characterization of the bond strength in both uniaxial tension and torsion. The following conclusions were drawn from the experimental results:

1. Hybrid PBF/wrought tensile failures occurred well away from the bond interface, in the wrought portion of the specimen, demonstrating that the interface bond quality was good. The torsion specimens also showed concentrated deformation in the wrought portion of each specimen.
2. The yield and ultimate tensile strengths of the SS316L PBF material were significantly greater than that of the wrought material of the same alloy (550 and 680 MPa for the PBF material, compared to 320 and 530 MPa for the wrought material).
3. Average grain sizes for the SS316L PBF material were 48 µm versus 60 µm for the wrought material. The Hall–Petch effect, which is pronounced in SS316L, is likely the reason for the greater strength of the PBF material and explains the behavior of the hybrid PBF/wrought specimens in uniaxial tension and torsion.

As both the PBF material and the bond interface exhibited strength equal to or greater than the original wrought material, using SS316L powder and PBF is a viable solution for printing new material onto existing structures, either for remanufacturing/repair or for creating new features. This observation complements similar research examining the PBF/wrought interface for maraging steels and titanium alloys [22,23]. As this technology is one of the most widely used AM processes for metals, it can be considered as an option in these applications when the desired interface is planar and can be positioned within the powder bed volume.

## Figures and Tables

**Table 1:** Summary of test specimens.

	SS316L Specimens		
	Wrought	PBF	PBF/Wrought
Tension	3	3	3
Torsion	2 ^1^	3	3
Microscopy	-	-	1

^1^ One additional SS316L wrought torsion specimen was not included due to testing irregularities.

**Table 2:** Measured tensile properties.

		Wrought	PBF	PBF/Wrought	ASTM Min. [25]	Typical [26]
$\sigma_{y\;}$	Mean	320 MPa	550 MPa	300 MPa	170 MPa	300 MPa
	StdDev	9.7 MPa	2.4 MPa	51 MPa		
$\sigma_{u}$	Mean	530 MPa	680 MPa	520 MPa	485 MPa	585 MPa
	StdDev	1.0 MPa	2.6 MPa	30 MPa		

**Table 3:** Statistical analysis of tensile specimens.

	Comparison	Statistical Test (α = 0.05)	Different? (*p* ≤ 5.0 × 10^−2^)
$\sigma_{y\;}$	Among group	ANOVA	Yes (*p* = 4.7 × 10^−4^)
	Wrought vs. PBF	*t*-test (equal variance)	Yes (*p* = 3.1 × 10^−5^)
	Wrought vs. PBF/Wrought	*t*-test (equal variance)	No (*p* = 5.4 × 10^−1^)
	PBF vs. PBF/Wrought	*t*-test (uneq. variance)	Yes (*p* = 1.4 × 10^−2^)
$\sigma_{U\;}$	Among group	ANOVA	Yes (*p* = 3.1 × 10^−4^)
	Wrought vs. PBF	*t*-test (equal. variance)	Yes (*p* = 6.2 × 10^−6^)
	Wrought vs. PBF/Wrought	*t*-test (uneq. variance)	No (*p* = 6.3 × 10^−1^)
	PBF vs. PBF/Wrought	*t*-test (uneq. variance)	Yes (*p* = 1.2 × 10^−2^)

**Table 4:** Measured torsion properties.

		Wrought	PBF	PBF/Wrought	Typical [26]
$\tau_{y\;}$	Mean	220 MPa	380 MPa	200 MPa	180 MPa
	StdDev	17 MPa	4.6 MPa	22 MPa	
G	Mean	71 GPa	70 GPa	61 GPa	77 GPa
	StdDev	3.7 GPa	0.4 GPa	5.5 GPa	

**Table 5:** Statistical analysis of torsion specimens.

	Comparison	Statistical Test (α = 0.05)	Different? (*p* ≤ 5.0 × 10^−2^)
$\tau_{y\;}$	Among group	ANOVA	Yes (*p* = 7.3 × 10^−5^)
	Wrought vs. PBF	*t*-test (equal variance)	Yes (*p* = 4.7 × 10^−4^)
	Wrought vs. PBF/Wrought	*t*-test (equal variance)	No (*p* = 4.3 × 10^−1^)
	PBF vs. PBF/Wrought	*t*-test (equal variance)	Yes (*p* = 5.0 × 10^−3^)
G	Among group	ANOVA	Yes (*p* = 4.9 × 10^−2^)
	Wrought vs. PBF	*t*-test (uneq. variance)	No (*p* = 7.4 × 10^−1^)
	Wrought vs. PBF/Wrought	*t*-test (equal variance)	No (*p* = 1.1 × 10^−1^)
	PBF vs. PBF/Wrought	*t*-test (uneq. variance)	No (*p* = 1.0 × 10^−1^)

**Table 6:** EBSD grain map analysis.

	Number of Grains	Average Grain Size	St. Dev. of Grain Size
Wrought	288	60 µm	13.64 µm
PBF	225	48 µm	14.31 µm

## Data Availability

Publicly available datasets were analyzed in this study. This data can be found here: https://scholarsarchive.byu.edu/data/25.

## Nomenclature

AM	Additive Manufacturing
DED	Directed Energy Deposition
EBSD	Electron Backscatter Diffraction
EDM	Electrical Discharge Machining
PBF	Powder Bed Fusion
